# Serum calcium is associated with sudden cardiac arrest in stroke patients from ICU: a multicenter retrospective study based on the eICU collaborative research database

**DOI:** 10.1038/s41598-023-51027-x

**Published:** 2024-01-19

**Authors:** Jianfei Hou, Zhenhua Huang, Wenfei Zeng, Zhanxing Wu, Lingna Zhang

**Affiliations:** 1 Department of Functional Examination, The First People’s Hospital of Chenzhou, Chenzhou, 423001 China; 2 Department of Emergency, Shenzhen Second People’s Hospital Shenzhen, Shenzhen, 518035 China; 3 Department of Anesthesiology, Hunan Provincial People’s Hospital, The First Affiliated Hospital of Hunan Normal University, Changsha, 410005 China

**Keywords:** Cardiology, Neurology

## Abstract

This primary objective of our study was to investigate the relationship between serum calcium levels and the occurrence of sudden cardiac arrest (SCA) in stroke patients. We analyzed the clinical data of 10,423 acute stroke patients admitted to the intensive care unit. The association between serum calcium and SCA following an acute stroke was assessed through multivariate logistic regression. We explored the non-linear connection between serum calcium levels and SCA in stroke patients using a generalized additive model and smooth curve fitting. Our study uncovered that serum calcium serves as an independent risk factor for sudden cardiac arrest in stroke patients. Notably, we observed that the relationship between serum calcium levels upon admission and the occurrence of SCA in stroke patients within the hospital was non-linear. Furthermore, we identified inflection points in serum calcium levels at 8.2 and 10.4 mg/dL. These findings emphasize a non-linear relationship between serum calcium levels and the risk of SCA in stroke patients. Maintaining serum calcium within the range of 8.2–10.4 mg/dL could lead to a significant reduction in the incidence of cardiac arrest among stroke patients.

## Introduction

Sudden cardiac arrest (SCA), also known as sudden death, is characterized by the sudden loss of arterial pulses, heart sounds, and severe ischemia and hypoxia in vital organs, ultimately leading to unexpected death^1^. SCA is a significant contributor to cardiovascular mortality, with an estimated 290,000 in-hospital cardiac arrests (IHCAs) occurring annually in the United States^2^. IHCAs are associated with high mortality rates and poor prognosis. Cardiac causes account for the majority of SCAs (50–60%), followed by respiratory insufficiency (15–40%)^3^. Stroke patients often experience various cardiovascular diseases and cardiac complications, including congestive heart failure (CHF), acute myocardial infarction (AMI), and abnormal heart rhythms such as tachyarrhythmia and bradyarrhythmia^4^. Even in patients without pronounced heart disease, 20–40% of stroke patients develop asymptomatic myocardial ischemia^5^. Acute stroke can disrupt central autonomic control, leading to myocardial injury, ECG abnormalities, arrhythmias, and ultimately, sudden death^6^. Therefore, the occurrence of SCA in stroke patients remains high and unpredictable, posing a significant challenge in identifying at-risk patients and preventing sudden death.

Calcium ions play a crucial role as signal transduction molecules in almost all cells, particularly in regulating cardiac physiology and electrophysiology^7,8^. Calcium ions play a crucial role as signal transduction molecules in almost all cells, particularly in regulating cardiac physiology and electrophysiology. While essential for cellular processes, improper control of calcium levels within cells can lead to serious dysfunctions and even cell death^9^. Although intracellular calcium’s role in cardiovascular physiology is well-established, the relationship between serum calcium levels and the development of arrhythmias and SCA is not well-defined.

The reference range for serum calcium falls within 8.9 to 10.1 mg/dL and is tightly regulated. Disturbances in calcium homeostasis can lead to various cardiovascular diseases. Hypercalcemia can result in QT interval prolongation, vascular calcification, and hypertension, while hypocalcemia can lead to life-threatening cardiac arrhythmias, cardiac arrest, heart failure, and prolonged QT intervals^8^. In Yarmohammadi et al.^10^ reported for the first time that low serum calcium levels are independently associated with an increased risk of SCA in the general population. Their multivariate analysis demonstrated a 2.3-fold increase in SCA risk for individuals with lower serum calcium levels compared to those with higher levels. Other studies have highlighted the link between dyscalcemia and the risk of cerebrovascular diseases, with hypocalcemia and hypercalcemia associated with an increased risk of stroke^11–13^. Additionally, serum calcium levels have been linked to infarct size, stroke outcomes, recurrence of ischemic stroke, and prognosis in stroke patients, suggesting that serum calcium may serve as a potential prognostic biomarker for stroke^14^.

A recent study also found a connection between serum calcium levels and the risk of stroke-associated infection (SAI), with the risk of SAI increasing as serum calcium levels fall below normal (9.0 mg/dL)^15^. Despite these findings, the value of serum calcium in predicting acute stroke-related SCA remains uncertain. Currently, blood calcium regulation in stroke patients relies heavily on the clinical experience of physicians. Therefore, this retrospective study aims to investigate the association between serum calcium levels and acute stroke-related SCA, offering valuable insights for the prevention and treatment of post-stroke cardiac arrest.

## Results

### Baseline characteristics

Table 1 and Supplementary Table 1 provide the demographic and clinical characteristics of the study participants. The average age was 67.07 ± 14.83 years. Among the 10,423 stroke patients, 5409 (51.9%) were male, and 5013 (48.1%) were female. During the study period, 201 (1.93%) patients experienced cardiac arrest in the hospital. The median serum calcium, ionized calcium, and albumin-corrected serum calcium (ACSC) were 8.85 ± 0.83 mg/dL, 9.63 ± 0.83 mg/dL, and 3.78 ± 1.57 mg/dL, respectively. We categorized participants into subgroups using tertiles, with T1 (serum calcium ≤ 8.5 mg/dL or ACSC ≤ 9.39 mg/dL), T2 (serum calcium 8.6–9.1 mg/dL or ACSC 9.4–9.95), and T3 (serum calcium ≥ 9.2 mg/dL or ACSC ≥ 9.96). Compared to T1, there were a significant differences in age, sex, ethnicity, Hb, PC, BUN, Mg, AF, CHF, ACS, COPD, cancer, or hypertension in T2 and T3 (P < 0.05). Additionally, in comparison to T1, hospital and ICU mortality rates were significantly reduced, and the length of stay in hospitals in T2 and T3 was also significantly shorter (P < 0.05). There were no significant differences in the type of stroke, cancer, or diabetes (P > 0.05).

**Table 1:** The baseline characteristics of participants.

Serum calcium (mg/dL)	T1 (< 8.5) N = 3086	T2 (8.6–9.1) N = 3721	T3 (> 9.2) N = 3616	P-value
Age (year)	66.25 ± 15.03	67.47 ± 14.74	67.36 ± 14.74	0.001
BMI (kg/m^2^)	26.7 (33.07, 31.4)	27.27 (23.30, 32.04)	27.45 (23.66, 31.98)	0.573
Sex				< 0.001
Male, n (%)	1697 (55.01%)	1988 (53.43%)	1724 (47.68%)	
Female, n (%)	1388 (44.99%)	1733 (46.57%)	1892 (52.32%)	
Ethnicity				< 0.001
African–American, n (%)	307 (10.00%)	433 (11.70%)	579 (16.10%)	
Asian, n (%)	65 (2.12%)	96 (2.59%)	74 (2.06%)	
Caucasian, n (%)	2274 (74.07%)	2750 (74.28%)	2643 (73.48%)	
Hispanic, n (%)	219 (7.13%)	179 (4.84%)	115 (3.20%)	
Native American, n (%)	25 (0.81%)	14 (0.38%)	8 (0.22%)	
Unknown, n (%)	180 (5.86%)	230 (6.21%)	178 (4.95%)	
AF, n (%)	415 (13.45%)	466 (12.52%)	382 (10.56%)	0.001
ACS, n (%)	171 (5.54%)	143 (3.84%)	130 (3.60%)	< 0.001
CHF, n (%)	202 (6.55%)	169 (4.54%)	145 (4.01%)	< 0.001
COPD, n (%)	149 (4.83%)	164 (4.41%)	119 (3.29%)	0.007
Diabetes, n (%)	410 (13.75%)	497 (13.36%)	431 (12.23%)	0.162
Hypertension, n (%)	922 (29.88%)	1239 (33.30%)	1137 (31.44%)	< 0.001
Cancer, n (%)	35 (1.13%)	28 (0.75%)	26 (0.72%)	0.129
Type of stroke				0.399
Hemorrhagic stroke, n (%)	988 (47.52%)	1163 (46.91%)	1148 (47.11%)	
Ischemic stroke, n (%)	1075 (51.71%)	1282 (51.71%)	1258 (51.62%)	
Others	16 (0.77%)	34 (1.37%)	31 (1.27%)	
Hb (g/L)	11.64 ± 2.37	12.88 ± 2.14	13.55 ± 2.06	< 0.001
PC (× 10^9^/L)	212.84 ± 88.35	226.09 ± 82.75	239.43 ± 90.05	< 0.001
BUN (mmol/L)	5.67 ± 3.04	5.34 ± 2.70	6.05 ± 3.08	0.254
Scr (mg/dL)	1.41 ± 1.52	1.18 ± 1.13	1.25 ± 1.21	< 0.001
Ionized calcium (mg/dL)	3.40 ± 1.63	3.88 ± 1.51	4.07 ± 1.50	< 0.001
Serum calcium (mg/dL)	7.95 ± 0.76	8.86 ± 0.17	9.60 ± 0.45	< 0.001
ACSC (mg/dL)	8.71 ± 0.73	9.62 ± 0.16	10.36 ± 0.48	< 0.001
Mg (mmol/L)	1.88 ± 0.39	1.91 ± 0.31	1.92 ± 0.34	< 0.001
GCS, median (Q1, Q3)	14.00 (10.00–15.00)	15.00 (11.25–15.00)	14.00 (11.00–15.00)	0.3275
Cardiac arrest	106 (3.43%)	44 (1.18%)	51 (1.41%)	< 0.001
Hospital mortality	547 (17.94%)	468 (12.75%)	415 (11.63%)	< 0.001
Hospital stay (day)	6.22 (3.26–12.16)	5.10 (2.92–9.59)	4.79 (2.75–8.57)	< 0.001
ICU mortality	321 (10.40%)	249 (6.69%)	229 (6.33%)	< 0.001
ICU stay (day)	2.38 (1.22–5.58)	1.96 (1.10–3.90)	1.90 (1.07–3.65)	< 0.001

Continuous variables are summarized as mean (SD) or median (quartile interval); categorical variables are presented as percentages (%).

*BMI* body mass index, *BUN* blood urea nitrogen, *Scr* serum creatinine, *RBC* red blood cell, *Hb* hemoglobin, *ACS* acute coronary syndrome, *AF* atrial fibrillation, *CHF* congestive heart-failure, *AMI* acute myocardial infarction, *COPD* chronic obstructive pulmonary disease, *ICU* intensive care unit, *PC* platelet count, *ACSC* albumin-corrected serum calcium.

### Stratification of stroke severity in stroke patients

**Figure 1:**
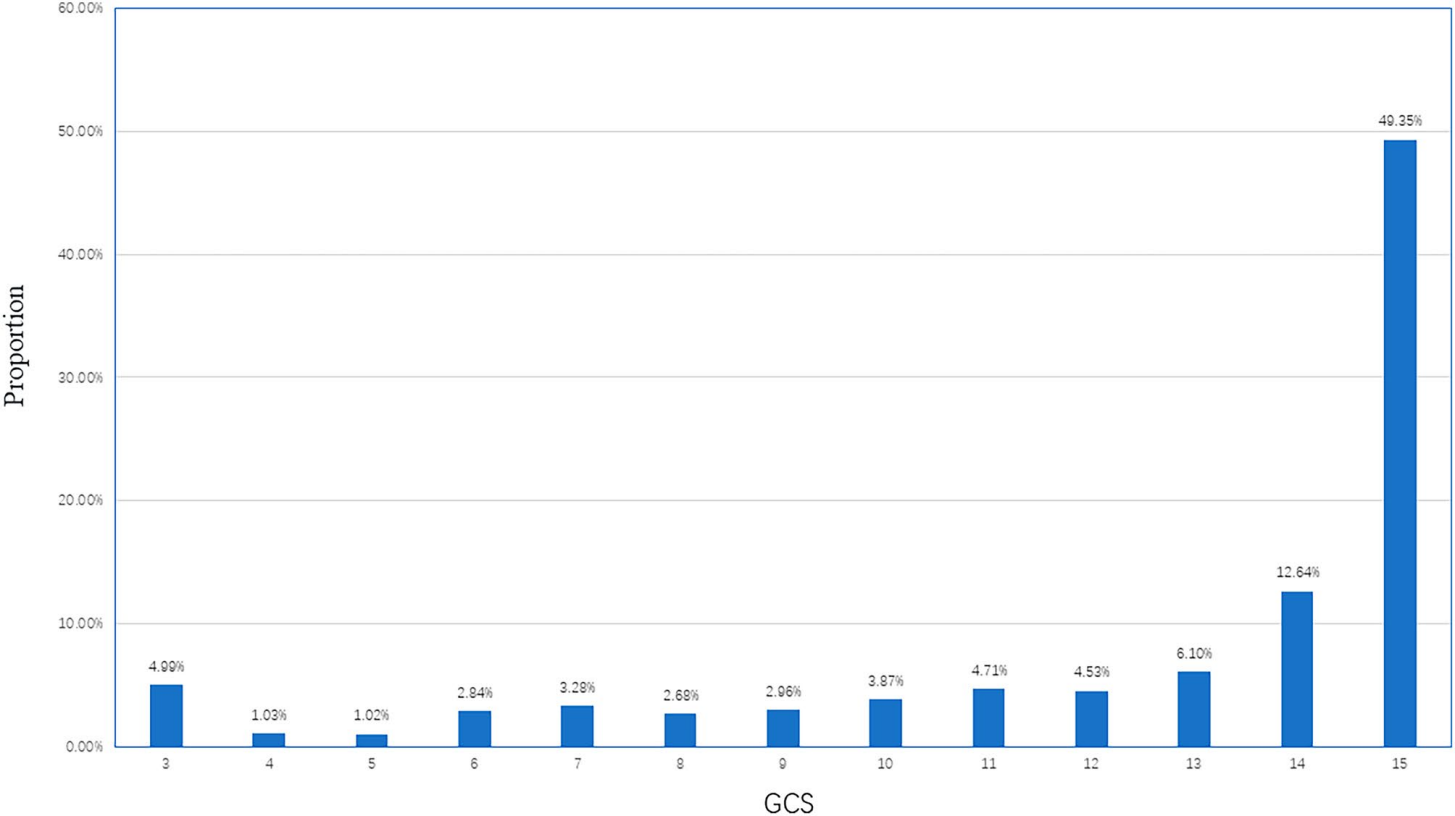
Distribution of GCS. It follows a non-normal distribution, ranging from 3 to 15.

**Figure 2:**
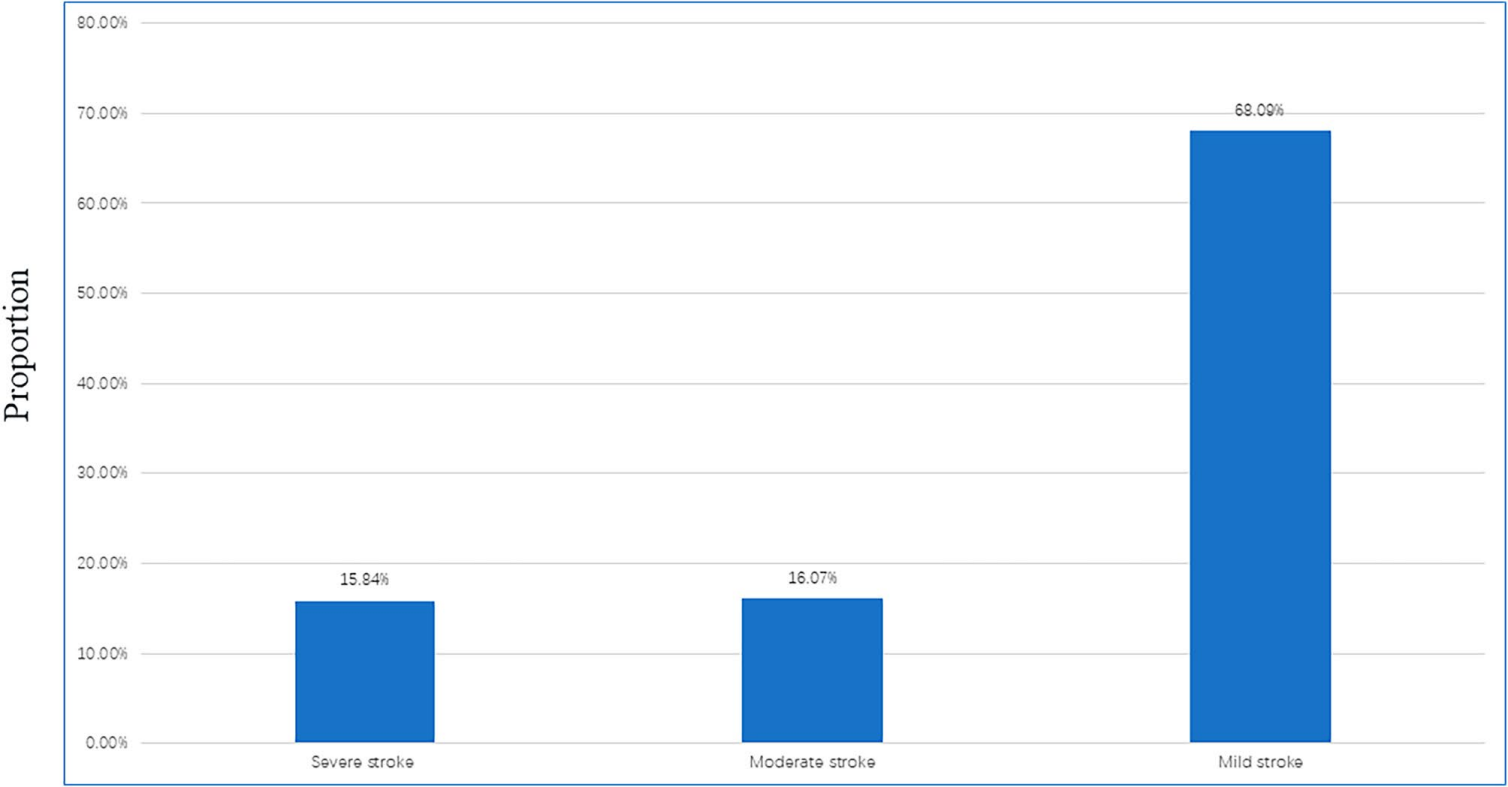
Stratification of stroke severity in stroke patients. As shown in the figure, severe strokes account for 15.84%, moderate strokes make up 16.07%, and the majority are mild cases, representing 68.09% of the total.

GCS follows a non-normal distribution, ranging from 3 to 15, with a median of 14 (Fig. 1). According to the GCS scores of stroke patients, we define GCS 13–15 as mild stroke with mild symptoms, GCS 9–12 as moderate stroke with moderate symptoms, and GCS 3–8 as severe stroke with severe symptoms, possibly accompanied by coma. Our results showed a severe stroke patient ratio of 15.8%. Most patients were classified as mild cases (Fig. 2).

### Factors influencing the risk of SCA in stroke patients by univariate analysis

As shown in Table 2, univariate analyses showed that serum calcium, ionized calcium and ACSC was associated with in-hospital SCA in stroke patients (*P* < 0.05). Similar results were found for age, levels of Hb, RBC, Scr, BUN, Mg, GCS, ACS, CHF, hypertension (all *P* < 0.05). However, in-hospital SCA was not associated with sex, BMI, AF, cancer, hypertension (all *P* > 0.05).

**Table 2:** Factors influencing risk of SCA in stroke patients analyzed by univariate analysis.

	Statistics	OR (95% CI)	P-value
Sex			
Male, n (%)	5409 (51.90%)	1.0	
Female, n (%)	5013 (48.10%)	0.83 (0.62, 1.11)	0.2137
Age (years)	67.07 ± 14.83	0.99 (0.98, 1.00)	0.0049
Ethnicity			
African–American, n (%)	1319 (12.72%)	1.0	
Asian, n (%)	235 (2.27%)	0.54 (0.19, 1.51)	0.2369
Caucasian, n (%)	7667 (73.94%)	0.48 (0.34, 0.70)	< 0.0001
Hispanic, n (%)	513 (4.95%)	0.99 (0.55, 1.78)	0.9684
Native American, n (%)	47 (0.45%)	0.67 (0.09, 4.95)	0.6911
Unknown, n (%)	588 (5.67%)	0.49 (0.24, 1.01)	0.0541
BMI (kg/m^2^)	334.22 ± 9454.25	1.00 (1.00, 1.00)	0.8592
AF, n (%)	1263 (12.12%)	1.33 (0.90, 1.96)	0.1484
ACS, n (%)	444 (4.26%)	5.25 (3.61, 7.63)	< 0.0001
CHF, n (%)	516 (4.95%)	1.92 (1.17, 3.14)	0.0094
COPD, n (%)	432 (4.14%)	2.33 (1.42, 3.82)	0.0008
Diabetes, n (%)	1369 (13.13%)	2.36 (1.71, 3.25)	< 0.0001
Hypertension, n (%)	3298 (31.64%)	0.87 (0.64, 1.19)	0.3914
Cancer, n (%)	89 (0.85%)	2.42 (0.88, 6.67)	0.0869
Hb (g/L)	12.75 ± 2.31	0.89 (0.84, 0.94)	0.0003
PC (× 10^9^/L)	226.78 ± 87.64	1.00 (1.00, 1.00)	< 0.0001
BUN (mmol/L)	22.01 ± 16.40	1.02 (1.01, 1.02)	< 0.0001
Scr (mg/dL)	1.27 ± 1.29	1.21 (1.15, 1.27)	< 0.0001
Serum calcium (mg/dL)	8.85 ± 0.83	0.65 (0.58, 0.74)	< 0.0001
Ionized calcium (mg/dL)	3.78 ± 1.57	0.86 (0.75, 0.99)	0.0308
ACSC (mg/dL)	9.63 ± 0.83	0.61(0.53, 0.69)	< 0.0001
Mg (mmol/L)	1.90 ± 0.35	1.85 (1.28, 2.67)	0.0010
GCS	12.5 ± 3.6	0.8 (0.8, 0.8 )	< 0.0001

### Relationship between serum calcium and SCA in stroke patients in different models

As shown in Table 3 and Supplementary Table 2, several risk factors including important clinical and significant variables in the univariate model were included in the multivariate model for adjustment. Serum calcium remained an independent predictor of in-hospital SCA in adjusted Model 1 (OR 0.65, 95% CI 0.58–0.74, P < 0.001) and Model 2 (OR 0.79, 95% CI 0.66–0.94, P < 0.001). Similarity, ACSC remained an independent predictor of in-hospital SCA in adjusted Model 1 (OR 0.61, 95% CI 0.53–0.70, P < 0.001) and Model 2 (OR 0.79, 95%CI 0.66–0.94, P = 0.0084).

**Table 3:** Relationship between serum calcium and cardiac arrest with stroke patients in different models.

Serum calcium	Non-adjusted model (OR, 95%, P)	Adjusted model I (OR, 95%, P)	Adjusted model II (OR, 95%, P)
Per 1-unit increase	0.65 (0.58, 0.74), < 0.0001	0.65 (0.58, 0.74), < 0.0001	0.79 (0.66, 0.94), 0.0084
(Tertile)			
T1	Ref	Ref	Ref
T2	0.34 (0.24, 0.48), < 0.0001	0.34 (0.24, 0.49), < 0.0001	0.47 (0.30, 0.74), 0.0010
T3	0.40 (0.29, 0.56), < 0.0001	0.40 (0.29, 0.57), < 0.0001	0.76 (0.49, 1.18), 0.2249
P for trend	< 0.0001	< 0.0001	0.1321

Non-adjusted model: no covariates were adjusted for.

Adjusted model I: we only adjusted for age, sex, and ethnicity.

Adjusted model II: we adjusted for age, sex, ethnicity, AF, CHF, ACS, COPD, diabetes, hypertension, GCS, Hb, Scr, and ALB.

### Non-linear relationship of serum calcium with SCA in stroke patients

**Figure 3:**
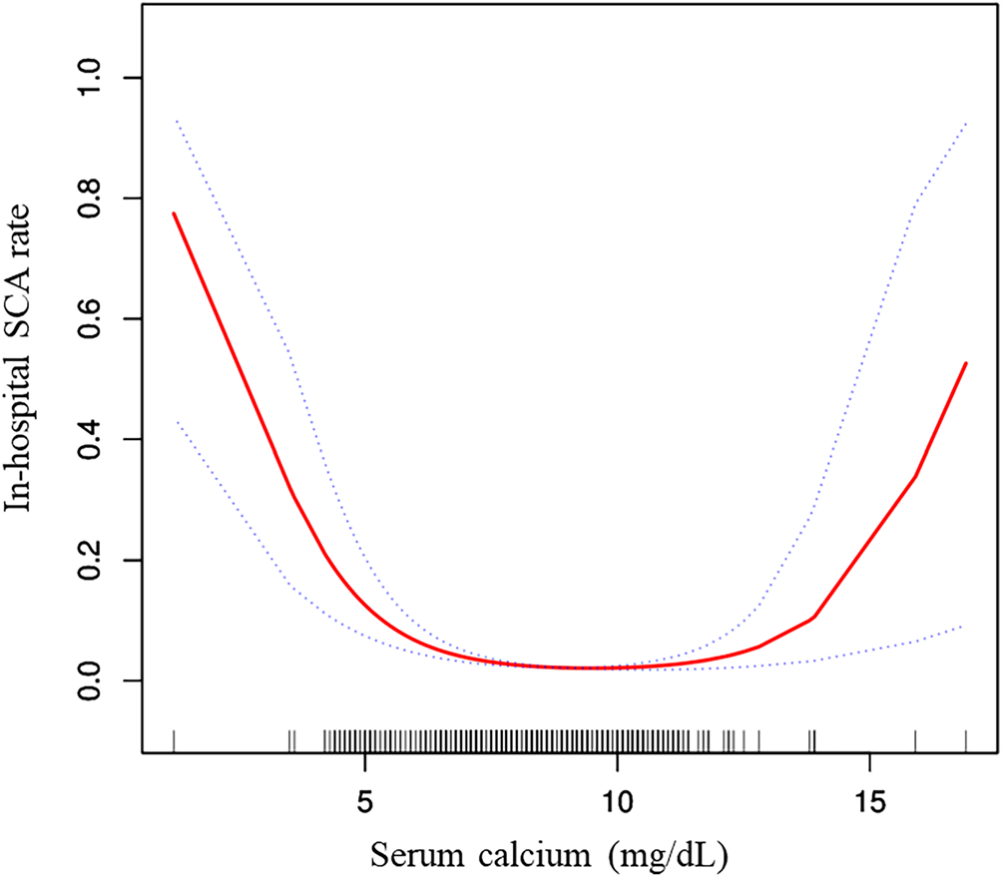
The nonlinear relationship of serum calcium with in-hospital SCA in stroke patients. The smoothing splines were a generated utilizing generalized additive model and adjusted for age, sex, ethnicity, AF, CHF, ACS, COPD, diabetes, hypertension, GCS, Hb, Scr, and ALB. The red line indicates the risk of cardiac arrest and the blue dotted line indicates the 95% confidence interval.

To further explore the relationship between serum calcium and the incidence of in-hospital cardiac arrest in stroke patients, we plotted the smoothing curves of serum calcium against the incidence of in-hospital cardiac arrest in stroke patients. Smoothing splines were generated utilizing a generalized additive model and adjusted for age, sex, ethnicity, AF, CHF, ACS, COPD, diabetes, hypertension, GCS, Hb, Scr, and ALB. The results showed that the link between serum calcium and the incidence of in-hospital cardiac arrest was nonlinear (U-shaped graph) (Fig. 3). In addition, we found that the inflection point of serum calcium was 8.2 and 10.4 mg/dL using a recursive algorithm. Then, we calculated the OR and CI on the left and right of the inflection point. The results showed that the OR was 0.53 on the left side of the 8.2 mg/dL (95% CI 0.24, 0.67) (P < 0.0001). In addition, when serum calcium was < 8.2 mg/dL, a 1-unit (× 1 mg/dL) decrease in serum calcium was associated with a 47% increase in the incidence of cardiac arrest in stroke patients. Interestingly, the OR was 2.37 on the right side of the 10.4 mg/dL (95% CI 1.58, 3.57) (P < 0.0001), a 1-unit (× 1 mg/dL) increase in serum calcium was associated with a 137% increase in the incidence of cardiac arrest in stroke patients (Table 4).

**Table 4:** The result of the two-piecewise linear regression model.

Stroke patients	OR, 95% CI	P
Inflection points of serum calcium (mg/dL) per 1-unit increase	8.2 and 10.4	
≤ 8.2	0.53 (0.42, 0.67)	< 0.0001
8.2–10.4	1.29 (0.99, 1.67)	0.0584
> 10.4	2.37 (1.58, 3.57)	< 0.0001
P-value for likelihood ratio test		< 0.0001

### Relationship of serum calcium to cardiac arrest according to type of stroke, sex, Scr and ESRD

As shown in Table 5, we conducted subgroup analysis to investigate potential additional risk factors that could influence the relationship between serum calcium and SCA in stroke patients. We examined the impact of gender, stroke type, renal function, and end-stage renal disease as stratification factors. However, our analysis revealed that males, ischemic stroke, creatinine levels, and end-stage renal disease all played significant roles in affecting the relationship between serum calcium and SCA in stroke patients.

**Table 5:** Stratified associations between serum calcium and SCA in patients with stroke by type of stroke, sex, Scr and ESRD.

Exposure	OR	95% (CI)	P-value
Type of stroke			
HS	0.71	(0.51, 1.00)	0.047
IS	0.67	(0.50, 0.88)	0.0043
Others	Inf.	(0.00, Inf)	0.9998
Sex			
Male	0.66	(0.53, 0.82)	< 0.0001
Female	0.81	(0.63, 1.05)	0.1153
Scr			
Low (0.1–0.8) (mg/dL)	1.02	(0.63, 1.63)	0.9458
Medium (0.8–1.1) (mg/dL)	0.60	(0.39, 0.91)	0.0173
High (1.1–21.2) (mg/dL)	0.80	(0.63, 1.00)	0.0471
ESRD			
Yes	0.58	(0.25, 1.33)	0.1964
No	0.79	(0.65, 0.95)	0.0121

*ESRD* end-stage renal disease.

## Discussion

This retrospective cohort study aimed to investigate the association between serum calcium and SCA in stroke patients. The main findings are as follows: Serum calcium and ACSC are independent risk factors for SCA occurrence in ICU stroke patients. This study observed, for the first time, a non-linear relationship between serum calcium and the risk of SCA during the hospitalization of stroke patients. We identified the inflection points for serum calcium as 8.2 mg/dL and 10.4 mg/dL. It is worth noting that maintaining serum calcium levels within the range of 8.2–10.4 mg/dL may significantly reduce the incidence of cardiac arrest in stroke patients. Subgroup analysis revealed that gender, ischemic stroke, creatinine levels, and end-stage renal disease all played significant roles in affecting the relationship between serum calcium and SCA in stroke patients.

Calcium homeostasis disorders can contribute to various cardiovascular diseases. Recent cohort studies have examined the connection between calcium levels and both cardiovascular and all-cause mortality. Interestingly, both high and low calcium levels have been linked to an increased risk of cardiovascular events. Both high and low levels of calcium were associated with an increase in cardiovascular events. Yamaguchi et al.^22^ identified hypocalcemia as a significant risk factor for mortality and cardiovascular events among incident hemodialysis patients. Similarly, Similarly, Miura and associates^23^ conducted a study that reviewed a group with low calcium levels, revealing significantly higher rates of cardiac and all-cause mortality, along with indications of heart and kidney damage in patients with heart failure and chronic kidney disease. Moreover, it was observed that lower calcium levels upon admission were associated with elevated in-hospital mortality in patients with ST-segment elevation myocardial infarction^24^. These findings align closely with the results of our study, where it is evident in Table 1 that patients with lower calcium levels (Q1) experienced higher in-hospital mortality.

In another study reported by Grandi et al., a cohort of patients with stable coronary artery disease (CAD) was followed for 8 years. They reported that patients with higher baseline serum calcium levels had significantly higher all-cause mortality. Grandi et al. suggested that higher calcium levels may contribute to the progression of vascular calcification and are associated with an increased risk of adverse cardiovascular events^25^. Additionally, Lundgren et al. reported that mild hypercalcemia was associated with premature death from cardiovascular disease^26^. In contrast to the studies, our study reached a different conclusion because the patients in our study hardly exceeded the upper limit of normal calcium levels. Furthermore, a recent study reported that lower serum calcium levels increased the risk of cardiovascular death in men and decreased the risk in women^27^. In our study, we also observed that male patients were more likely to experience SCA. Additionally, we found that the type of stroke and kidney function status could also influence the relationship between serum calcium and SCA in stroke patients.

Yarmohammadi et al.^10^ found that low serum calcium levels are independently associated with an increased risk of sudden cardiac arrest (SCA) in the general population. For each decrease of one unit in calcium, there was a 1.6-fold increase in the odds of SCA (OR 1.63, 95% CI 1.06–2.51). This is in line with our study findings. However, their study specifically recruited high-risk groups for cardiac disease in both the experimental and control groups, leading to more than 80% of individuals in the “general population”, in both groups, having coronary heart disease. Furthermore, their study included some patients with renal insufficiency and those undergoing hemodialysis. Therefore, their study results may not fully represent all population groups, especially hospitalized stroke patients. Hypocalcemia is extremely common in hospitalized patients (up to 88%) and correlates with the severity of the disease^28^. Stroke, with higher mortality, is a critical illness, that often has a variety of cardiovascular diseases and cardiac complications. Due to increasingly severe conditions, an increasing proportion of acute stroke patients are being admitted to an ICU^29^. It is obvious that stroke patients may also have hypocalcemia. Hence, further investigating the relationship between serum calcium and SCA may be favorable for stroke patients.

The results of this study are consistent with a previous study^10^. This study demonstrated that baseline serum calcium levels were associated with the occurrence of sudden cardiac arrest (SCA) in patients with acute stroke. When serum calcium levels were categorized into tertiles, our results showed that the group with the lowest serum calcium levels had a significantly increased incidence of cardiac arrest. The trend in effect sizes between the groups was equidistant when serum calcium was treated as a categorical variable. The trend P-value was consistent with the result when serum calcium was analyzed as a continuous variable. This finding is also in line with a recent study^11^. However, previous studies did not specify whether the relationship between serum calcium and cardiac arrest was linear or nonlinear, and the study population was the general population, limiting the significance of the findings. In this study, we used a generalized additive model (GAM) and smooth curve fitting to demonstrate a nonlinear relationship between serum calcium and the risk of SCA in stroke patients. After adjusting for confounding factors, the inflection point of serum calcium was determined to be 8.2 mg/dL and 10.4 mmol/L. When serum calcium was below 8.2 mg/dL or above 10.4 mg/dL, the risk of SCA in stroke patients was significantly increased. This finding provides valuable guidance for the prevention of SCA in ICU patients.

This study has some notable strengths. First, the reliability of the data benefits from the relatively large sample size, as it is a multicenter study covering patient data from 208 hospitals in the United States. Second, we observed a nonlinear relationship between serum calcium and the risk of in-hospital sudden cardiac arrest (SCA) in stroke patients. Additionally, we found that maintaining serum calcium within the range of 8.2–10.4 mg/dL may significantly reduce the incidence of cardiac arrest in stroke patients.

However, this study also has some potential limitations. Firstly, it is a retrospective analysis study, which may not eliminate potential confounding factors. Secondly, the database used for this study only recorded serum calcium levels at the time of admission and did not provide data for multiple measurements. Nevertheless, future research will focus on studying the impact of changes in serum calcium on the occurrence of sudden cardiac arrest in stroke patients. Furthermore, all patients included in this study came from intensive care units, so this conclusion may not be applicable to patients in general wards. Additionally, due to the database providing only short-term follow-up data, we cannot determine long-term outcomes. Finally, the relationship between serum calcium and the risk of in-hospital SCA in stroke patients may not necessarily be causal, and longitudinal analysis is needed to reveal more causative insights.

## Conclusions

Serum calcium was an independent risk factor for the incidence of SCA poststroke in critically ill stroke patients. For the first time, we report a nonlinear relationship between serum calcium levels and in-hospital SCA risk in stroke patients. Maintaining serum calcium levels within the range of 8.2–10.4 mg/dL may significantly reduce the incidence of cardiac arrest in stroke patients.

## Methods

### Data source

The data analyzed in this study were extracted from the eICU-CRD, a multicenter intensive care unit database containing over 200,000 cases^16,17^. As a multicenter database, the e-ICU platform documents the electronic medical records of patients from 208 hospitals in the United States from 2014 to 2015. The use of this database was approved by the Institutional Review Board of the Massachusetts Institute of Technology (Cambridge, Massachusetts, USA). One author (Zhenhua Huang) obtained access rights and was responsible for data extraction (certification number: 49995491). All methods were performed in accordance with the eICU-CRD relevant guidelines and regulations.

### Study population

**Figure 4:**
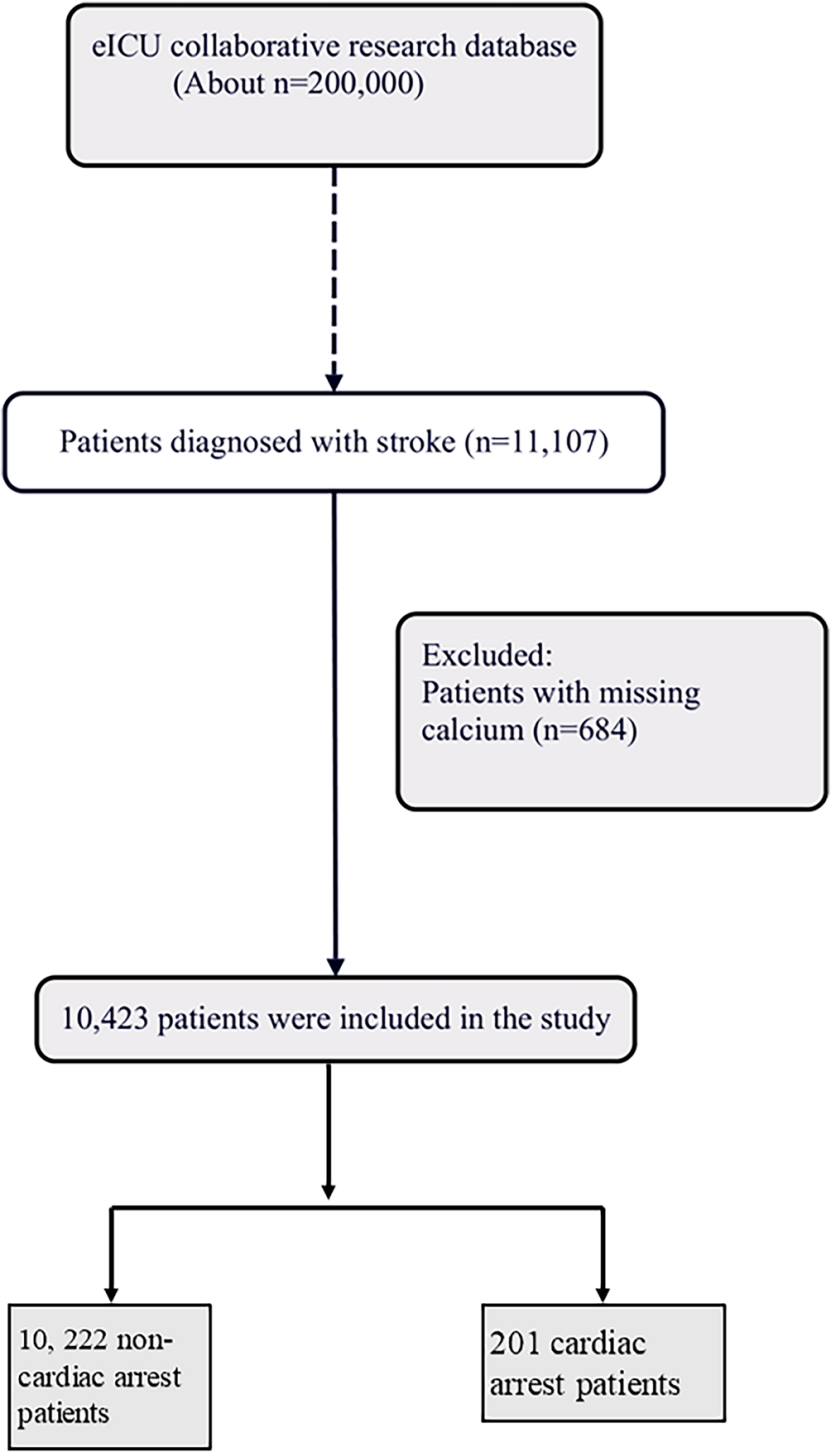
Flowchart of study participants.

Patients with a primary diagnosis of brain stroke, were grouped into 3 categories: ischemic stroke (IS) group, hemorrhagic stroke (HS) group, and others group^18^. Sudden cardiac arrest (SCA) was defined as a sudden unexpected pulseless condition and noncardiac etiologies^19^. All patients included were admitted for stroke. All enrolled patients signed an informed consent form, according to the World Medical Association Declaration of Helsinki, revised in 2013. Cardiac arrest occurs during hospitalization. This study initially included 11,107 patients diagnosed with stroke. Subsequently, 684 patients were excluded for missing calcium. Ultimately, 10423 stroke patients were included. Of them, 201 patients suffered cardiac arrest (Fig. 4).

### Demographic and laboratory variables

All data were extracted using Structured Query Language (SQL) before further analysis. The data of interest to this present study included sex, age, ethnicity, admission height, admission weight, BMI, stroke types, the status of discharge hospital or ICU, atrial fibrillation (AF), congestive heart failure (CHF), acute coronary syndrome (ACS), acute myocardial infarction (AMI), hypertension, diabetes mellitus, chronic obstructive pulmonary disease (COPD), blood urea nitrogen (BUN), serum creatinine (Scr), red blood cell (RBC), hemoglobin (Hb), platelet count (PC), and so on.

The calculation of albumin-corrected serum calcium (ACSC) was based on a standard formula and epidemiological data from the northern European population^20^.

The calculation formula is as follows: ACSC = measured serum calcium level + 0.020 × (41.3 − serum albumin), where 41.3 g/L represents the median albumin^21^. The units of ACSC and serum calcium have been converted to milligrams per deciliter (mg/dL). If serum calcium and some variables were measured several times after ICU entry, data from the first time were used. The unit of serum calcium was mg/dL. The primary study outcome was in-hospital SCA in stroke patients.

### Statistical analysis

Frequency and percentages were used for categorical variables. Continuous variables were first assessed for normality. Normal data are expressed as the mean and standard deviation (SD) and were compared using Student’s t test or one-way ANOVA. Nonnormal data are expressed as the median with interquartile range (IQR) and were compared using the Wilcoxon rank-sum test. Variables with a two-tailed P value < 0.05 were statistically significant and were included in the regression model. Multivariate-adjusted odds ratios (OR) and 95% confidence intervals (CI) for the study outcomes and calcium (1 unit and tertiles) were calculated by logistic regression analysis. Following covariate screening, we built three different models using both univariate and multivariate binary logistic regression models to study the relationship between serum calcium and in-hospital SCA poststroke. The model was as follows: (i) nonadjusted model (unadjusted covariates); (ii) minimum adjustment model (Model I: adjusted for age, sex, and ethnicity); (iii) fully adjusted model (age, sex, ethnicity, AF, CHF, ACS, COPD, diabetes, hypertension, GCS, Hb, Scr, and ALB). All P values were two-tailed and a P value < 0.05 was considered statistically significant. The smoothing curves and forest plots were illustrated by EmpowerStats (X&Y Solutions, Inc., Boston, MA).

### Supplementary Information

Supplementary Tables.

## Data Availability

The data used to support the findings of this study are available from the corresponding author or the first author upon request.
