# Supplementary material for: Serum calcium is associated with sudden cardiac arrest in stroke patients from ICU: a multicenter retrospective study based on the eICU collaborative research database
Source: Sci Rep. 2024 Jan 19;14:1700. doi: 10.1038/s41598-023-51027-x (PMC10799080; doi:10.1038/s41598-023-51027-x)
Supplement: Supplementary file 1 — Supplementary Tables. [file 41598_2023_51027_MOESM1_ESM.docx]

Supplementary Table 1. The baseline characteristics of participants.

| ACSC (mg/dL) | T1 (＜9.39)  N=2490 | T2 (9.4-9.95)  N=2485 | T3 (＞9.96)  N=2495 | P-value |
| --- | --- | --- | --- | --- |
| age（year） | 65.84 ± 14.89 | 67.54 ± 14.53 | 67.93 ± 14.44 | <0.001 |
| BMI (kg/m 2) | 26.78 (23.22-31.62) | 27.27 (23.25-31.84) | 27.43 (23.68-31.95) | 0.481 |
| Sex |  |  |  | <0.001 |
| Male, n (%) | 1405 (56.43%) | 1304 (52.47%) | 1185 (47.49%) |  |
| Female, n (%) | 1085 (43.57%) | 1181 (47.53%) | 1310 (52.51%) |  |
| Ethnicity |  |  |  | <0.001 |
| African-American, n (%) | 244 (9.85%) | 278 (11.27%) | 403 (16.22%) |  |
| Asian, n (%) | 54 (2.18%) | 71 (2.88%) | 49 (1.97%) |  |
| Caucasian, n (%) | 1825 (73.65%) | 1832 (74.26%) | 1824 (73.43%) |  |
| Hispanic, n (%) | 182 (7.34%) | 115 (4.66%) | 81 (3.26%) |  |
| Native American, n (%) | 17 (0.69%) | 10 (0.41%) | 5 (0.20%) |  |
| Unknown, n (%) | 156 (6.30%) | 161 (6.53%) | 122 (4.91%) |  |
| AF, n (%) | 365 (14.66%) | 284 (11.43%) | 266 (10.66%) | 0.001 |
| ACS, n (%) | 171 (6.87%) | 91 (3.66%) | 101 (4.05%) | <0.001 |
| CHF, n (%) | 172 (6.91%) | 110 (4.43%) | 107 (4.29%) | <0.001 |
| COPD, n (%) | 116 (4.66%) | 107 (4.31%) | 88 (3.53%) | 0.007 |
| Diabetes, n (%) | 378 (15.18%) | 306 (12.31%) | 322 (12.91%) | 0.162 |
| Hypertension, n (%) | 756 (30.36%) | 774 (31.15%) | 746 (29.90%) | <0.001 |
| carcer, n (%) | 31 (1.24%) | 16 (0.64%) | 23 (0.92%) | 0.129 |
| Type of stroke |  |  |  | 0.236 |
| Hemorrhagic stroke, n (%) | 796 (48.21%) | 733 (44.61%) | 801 (47.96%) |  |
| Ischemic stroke, n (%) | 836 (50.64%) | 889 (54.11%) | 847 (50.72%) |  |
| others | 19 (1.15%) | 21 (1.28%) | 22 (1.32%) |  |
| Hb (g/L) | 11.78 ± 2.43 | 13.03 ± 2.18 | 13.55 ± 2.11 | <0.001 |
| PC (× 109/L) | 213.43 ± 89.55 | 229.43 ± 85.06 | 239.99 ± 90.21 | <0.001 |
| BUN (mmol/L) | 25.28 ± 20.80 | 20.92 ± 14.34 | 22.96 ± 16.41 | <0.001 |
| Scr (mg/dl) | 1.52 ± 1.68 | 1.23 ± 1.16 | 1.32 ± 1.30 | <0.001 |
| Ionized calcium (mg/dL) | 3.38 ± 1.65 | 3.82 ± 1.53 | 4.06 ± 1.50 | <0.001 |
| Serum valcium (mg/dL) | 8.04 ± 0.72 | 8.94 ± 0.16 | 9.65 ± 0.48 | <0.001 |
| ACSC (mg/dL) | 8.80 ± 0.72 | 9.69 ± 0.15 | 10.40 ± 0.48 | <0.001 |
| Mg (mmol/L) | 1.88 ± 0.40 | 1.91 ± 0.32 | 1.93 ± 0.35 | 0.002 |
| Cardiac arrest | 106 (3.43%) | 44 (1.18%) | 51 (1.41%) | <0.001 |
| Hospital mortality | 489 (19.86%) | 311 (12.73%) | 317 (12.89%) | <0.001 |
| Hospital stays (day) | 10.87 ± 18.27 | 8.74 ± 15.73 | 7.93 ± 10.32 | <0.001 |
| ICU mortality | 291 (11.69%) | 180 (7.24%) | 175 (7.01%) | <0.001 |
| ICU stays (day) | 5.40 ± 7.29 | 3.94 ± 5.47 | 3.71 ± 4.95 | <0.001 |

Continuous variables are summarized as mean (SD) or median (quartile interval); categorical variables are presented as percentages (%).

BMI, body mass index, BUN, blood urea nitrogen; Scr, serum creatinine; RBC, red blood cell; Hb, hemoglobin; ACS, acute coronary syndrome; AF, atrial fibrillation; CHF, congestive heart-failure, AMI, acute myocardial infarction; COPD, chronic obstructive pulmonary disease; ICU, intensive care unit; PC, platelet count. ACSC, albumin-corrected serum calcium.

Supplementary Table 2. Relationship between ACSC and SCA with stroke patients in different models

|  | Non-adjusted model  (OR,95%, P) | Adjusted model I  (OR,95%, P) | Adjusted model II  (OR,95%, P) |
| --- | --- | --- | --- |
| ACSC (mg/dL) | 0.61 (0.53, 0.69) <0.0001 | 0.61 (0.53, 0.70) <0.0001 | 0.79 (0.66, 0.94) 0.0084 |
| Mg (mmol/l) | 1.85 (1.28, 2.67) 0.0010 | 1.85 (1.28, 2.68) 0.0011 | 1.37 (0.92, 2.03) 0.1219 |
| Ionized calcium (mg/dL) | 0.86 (0.75, 0.99) 0.0308 | 0.88 (0.77, 1.02) 0.0909 | 1.07 (0.88, 1.29) 0.5199 |

Non-adjusted model: no covariates were adjusted for.

Adjusted model I: we only adjusted for age, sex, and ethnicity.

Adjusted model II: we adjusted for age, sex, ethnicity, AF, CHF, ACS, COPD, diabetes, hypertension, GCS, Hb, Scr, and ALB.
